# Three-Dimensional Analysis of Biomimetic Aerofoil in Transonic Flow

**DOI:** 10.3390/biomimetics7010020

**Published:** 2022-01-22

**Authors:** Siva Marimuthu, Samer Al-Rabeei, Hithim Ahmed Boha

**Affiliations:** 1 Department of Engineering, Staffordshire University, Stoke-on-Trent ST4 2DE, UK; 2 Technical University of Košice, 040 01 Košice, Slovakia; samer.al-rabeei@tuke.sk (S.A.-R.); amtokal@hotmail.com (H.A.B.)

**Keywords:** aerofoil, biomimetics, computational fluid dynamics, transonic flow, viscous reduction

## Abstract

Since the invention of the aircraft, there has been a need for better surface design to enhance performance. This thirst has driven many aerodynamicists to develop various types of aerofoils. Most researchers have strongly assumed that smooth surfaces would be more suitable for air transport vehicles. This ideology was shattered into pieces when biomimetics was introduced. Biomimetics emphasized the roughness of a surface instead of smoothness in a fluid flow regime. In this research, the most popular 0012 aerofoils of the National Advisory Committee for Aeronautics (NACA) are considered to improve them, with the help of a surface pattern derived from the biological environment. Original and biomimetic aerofoils were designed in three dimensions with the help of Solidworks software and analyzed in the computational flow domain using the commercial code ANSYS Fluent. The implemented biomimetic rough surface pattern upgraded the NACA 0012 aerofoil design in the transonic flow regime. Lift and viscous forces of the aerofoil improved up to 5.41% and 9.98%, respectively. This research has proved that a surface with a little roughness is better than a smooth surface.

## 1. Introduction

In past decades, the rapid increase in people’s interest in aviation has influenced the entire aircraft industry to frequently improve itself in all aspects. Owing to that, many researchers, academics, and industrialists are trying very hard to develop new methodologies in order to meet current demands. The urgency to reduce carbon emissions in aviation is essential. With the pledges made in the recent Climate Change Conference held in Glasgow, the necessity to keep global warming within 1.5 degrees is of international importance. Humankind is under threat due to the increasing rate of climate change. The United Kingdom has included international aviation in their current sixth carbon budget which emphasizes the requirement of alternate solutions in the field of aviation. This has influenced all areas of the aerospace industry. As a part of this renovation process, most NACA aerofoils have been redesigned in order to improve their performance. NACA has three common classifications of the aerofoil: four-digit, five-digit, and six-digit aerofoils. In this computational analysis, NACA 0012 aerofoil is taken into account. The last two digits of this four-digit aerofoil denote a 12% chord of maximum thickness. Although NACA 0012 is used extensively in low and medium subsonic flows, it is not effective at high subsonic Mach numbers which are also called transonic flow.

A lot of commercial aircraft such as Airbus A320, Boeing 737, and so on, are operated within the transonic speed range. These commercial aircraft contribute highly to carbon emissions, although many steps have been taken by the manufacturers to reduce the emission in the transonic flow. It is still considered a challenging issue. Transonic flow is a combination of subsonic and supersonic Mach numbers. The range is between 0.8 and 1.2 Mach. NACA 0012 undergoes an abrupt upsurge in drag coefficient with velocity approaching the sonic velocity [1]. The viscous forces are high in this situation. This rapid change has urged many researchers to investigate the performance of NACA 0012 aerofoil in transonic flow. After several attempts, this issue has not yet been fixed. The main objectives of this research are to (i) reduce viscous drag, (ii) enhance lift, (iii) develop a biomimetic pattern, and (iv) emphasize rough surface in transonic flow over an aerofoil. In this research, the three-dimensional design of NACA 0012 is analyzed in airflow at 0.8 Mach with the use of computational fluid dynamics. The irregularities in the flow are solved with a help of a rough surface pattern called ‘Raw Riblet’ obtained from shark skin. Based on the mimicking concepts, a biomimetic aerofoil is made with enhanced performance in high subsonic flow when compared to NACA 0012.

## 2. Literature Review

Generally, aerofoils tend to experience two types of drags in fluid flow, i.e., pressure and viscous drags. When fluid flows over a surface, the distance between the fluid molecules and the surface will get reduced, which will increase the collision between them. This interaction between the wall and fluid is called surface drag, which is also referred to as viscous drag [2]. Skin friction drag is very dominant in a transonic fluid flow over an aerofoil. In order to reduce this viscous drag, various methodologies have been tried by several researchers. Rasuo, [3] studied the fluid parameters such as Mach and Reynolds numbers and wall interference with reference to solid and blockage of wake as well as the influence of side-wall boundary layer control. These theoretical and experimental studies on NACA 0012 aerofoil have given us the idea of performance factors that affect the wind tunnel results. Therefore, this research is focused on an aerofoil’s surface which influences the flow.

In order to modify the smooth surface of NACA aerofoil, a biomimetic pattern is considered. Biomimetics is a method of duplicating solutions from nature. For example, the dragonfly aircraft [4] was designed based on the wing planform of a dragonfly. It led to turbulence reduction when biomimetics emphasized roughness instead of smoothness to reduce turbulence [5]. This was proved in internal computational studies [6] and also in external computational studies [7]. Sharks such as requiem, hammerhead, and nurse sharks have peculiar skin patterns that could modify a smooth surface. Feld et al., [8] studied the dermal denticles of various spark species in their research. Techniques such as micro-fluid experiments and numerical simulations were used in their investigation which resulted in finding negative flow effects such as vortices and bubbles. The shark species used in their research were generally of smaller size and slow speed. Based on this study, we can narrow down our focus to large shark species which will be expected to have skin texture with higher flow advantages when compared to the smaller sharks. Lang et al., [9] carried out their research on the shortfin mako (Isurus oxyrinchus), considered to be one of the fastest and most agile marine predators, is known to have highly flexible scales on certain locations of its body. Brian and Bharat, [2] identified that the skin of fast-swimming sharks exhibits riblet structures aligned in the direction of flow that is known to reduce skin friction drag in the turbulent-flow regime. Bechert et al., [10] noticed that scales with V-shaped central ridges are good in solving fluid problems and are present on the surface of pectoral fins of a number of shark species. Bhatia et al., [11] improved the aerodynamic efficiency of a NACA 0012 aerofoil with the use of dermal denticles obtained from shark skin. In their research, two different shapes of denticles were designed and placed along the chord line of the aerofoil. This design was analyzed computationally in two dimensions which resulted in reduced drag at different angles of attack for different locations of the sharkskin denticles. However, this was not done in three dimensions and the values were not validated with experimental results.

Although many types of research have been done on shark skin, a nonlinear spatiotemporal analytical model is not available that explains the mechanism underlying control of flow with such proud patterns, despite the fact that shark skins are major targets of reverse engineering mechanisms of drag reduction [12]. Liu et al., [13] reviewed more than fifty research articles related to shark skin for drag reduction in fluid flow. They concluded at the end of their research review that the effect of drag reduction is obvious, but it still has a long way to go both in theoretical and practical application. They observed a great drag reduction rate under certain conditions and noticed that the drag reduction effect would be weakened or disappear if the conditions are changed. They added that many issues in turbulence and engineering applications have not yet been solved and require a functional relationship between the drag reduction effect and bioinspired surface. Hence, after careful consideration, a requiem shark skin pattern was chosen for its effectiveness in fluid flow. Therefore, in this transonic flow analysis over the NACA 0012 aerofoil, a requiem shark skin surface pattern is designed and implemented on the smooth aerofoil surface to roughen it and reduce friction or viscous drag with the help of computational tools. Computational fluid dynamics (CFD) incorporates mathematical relations and algorithms to analyze and solve problems regarding fluid flow [14]. Wan et al., [15] implemented numerical computations to investigate the aerodynamic characteristics of a NACA 0012 airfoil in transonic flows at various angles of attack and obtained promising results. Rabii et al., [14] investigated NACA 0012 and they predicted that lift, drag, and pressure coefficients are in good agreement with experimental data. Novel and Shadab, [16] identified in their transonic compressible flow simulation that NACA 0012 aerofoil stalls at a 16° angle of attack. This shows the necessity to enhance the performance of NACA 0012 aerofoil in transonic flow.

## 3. Computational Procedure

**Figure 1:**
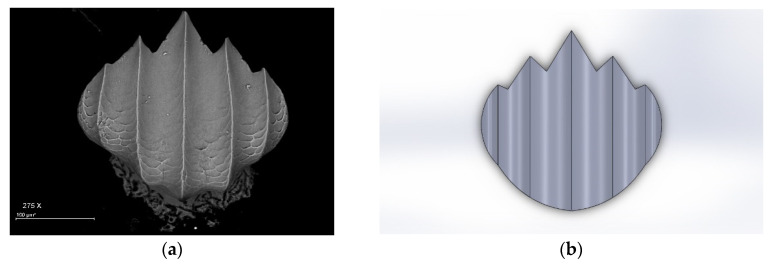

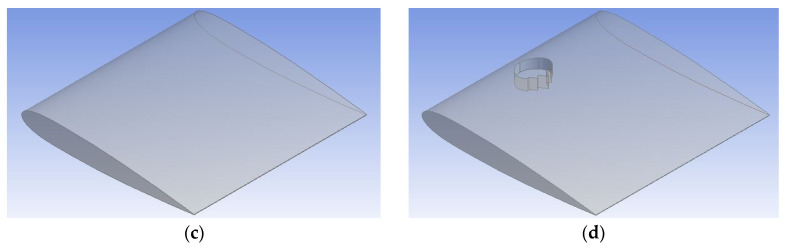
Aerofoil computational design. (**a**) Requiem shark skin’s SEM image. Adapted from [18]. (**b**) Raw riblet pattern. (**c**) NACA 0012 aerofoil. (**d**) 1RR HPBA.

Sharkskin patterns are made from three common families. They are requiem, hammerhead, and nurse sharks. Shark scales display a wide variation in geometry both across species while also varying with body location [17]. Out of these varieties, a requiem shark was chosen because of its non-complicated shape and its effectiveness. The dimensions of the skin pattern were optimized from the scanning electron microscopic (SEM) image of the requiem shark as shown in Figure 1a. With the help of lengths, breadths, and heights from the microscopic image, a raw riblet pattern (RR) was made in three dimensions using Solidworks software as shown in Figure 1b. Similarly, a NACA 0012 aerofoil was designed in Solidworks with the use of dimensions obtained from airfoiltools.com as shown in Figure 1c. The designed raw riblet pattern was implemented on the top surface of NACA 0012 aerofoil with the help of an extrude-cut feature in Solidworks. This hollow raw riblet pattern was placed exactly at the center line of the top surface near the leading edge and was cut to the mid-surface of the aerofoil as shown in Figure 1d. Due to the implementation of one raw riblet hollow biomimetic pattern on NACA aerofoil, this new model was named ‘1-RR HPBA’.

The three-dimensional designs of NACA and biomimetic aerofoils were imported into ANSYS simulation software. Aerofoils were split into the top, bottom, and riblet surfaces (in 1RR HPBA) with the use of a face-split tool in the design modeler. Additionally, a control volume was generated with appropriate dimensions required for transonic flow. Boundary conditions like pressure far-field and wall sections were set with the help of the name selection tool. This would enable the required properties in computational analysis. Computational domain and aerofoil were defined as target and tool bodies, respectively. The defined design meshed with default values in order to divide it into small units of cells also called grids. This would enable the commercial code to solve the governing equations of fluids effectively. The number of nodes and elements were noted.

**Figure 2:**
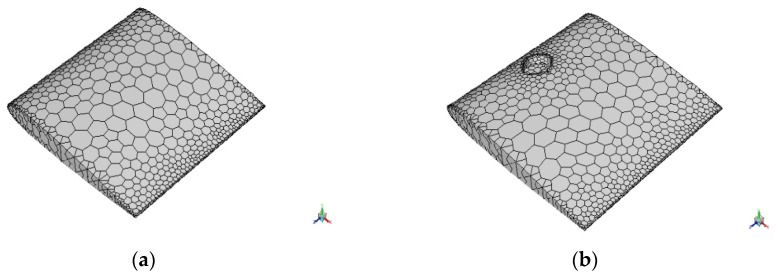

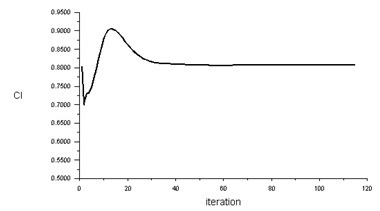
Computational analysis. (**a**) Polyhedral mesh of NACA 0012. (**b**) Polyhedral mesh of 1RR HPBA. (**c**) Iteration curve of 1RR HPBA.

The meshed models were brought into Fluent and converted into a polyhedral mesh as shown in Figure 2a, which would reduce the computational cost and time. It was then checked for scale and reported the quality of the mesh. The spalart allmaras (SA) viscous model was chosen for its effectiveness in aerodynamic flows. SA is also called a one equation model because it solves the eddy viscosity equation in addition to the Navier stokes equation. The inlet velocity of fluid was set to 0.8 Mach (transonic flow). Turbulence intensity and viscosity values were set to 1 and 5, respectively. The properties of air were set to default. The Gauge and operating pressures were set to 0 and 66471 pascals, respectively. This was calculated based on the physical properties of air and Mach number. Coupled solution method and second-order upwind were chosen in order to obtain high accuracy at a lower computational time. The eulerian approach was used which would help to solve problems individually and also enable the modification of flow angle instead of an aerofoil. NACA 0012 and 1-RR HPBA models were initialized, checked for errors, and iterated at 0°, 3°, 6°, 9°, 12°, 15°, 18°, 21°, 24°, 27°, and 30° angles of attack. Results were obtained when the solution converged. For example, the calculation converged as shown in Figure 2b, which is a lift iteration curve of 1RR HPBA at 27° angles of attack and converged at the 115th iteration.

## 4. Results and Discussion

### 4.1. Lift Force

**Figure 3:**
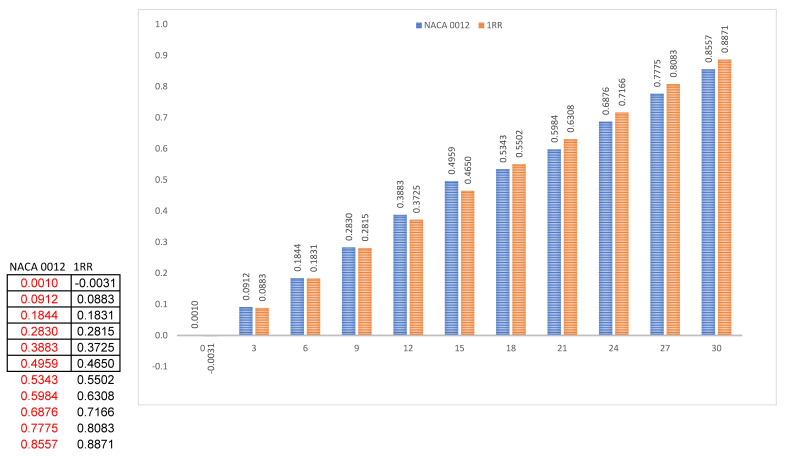
Lift analysis. (**a**) Pressure pathlines of NACA 0012. (**b**) Pressure pathlines of 1RR HPBA. (**c**) Lift generated at all angles of attack. (**d**) Lift increment in percentage.

Figure 3a clearly shows the pressure distribution on the top surface of the NACA 0012 aerofoil. This shows that NACA has above medium values of pressure which start near to the leading edge and cover a maximum area on the top surface. Generally, an aerofoil requires lower values of pressure on the top surface as indicated in Figure 3b, in order to generate higher values of lift. Pressure has been reduced to a medium value with the use of the RR pattern. At low and medium angles of attack i.e., from 0° to 15°, the values of lift generated are higher in NACA 0012 when compared to 1RR HPBA, whereas from 18° and above, the values of lift increased drastically in the 1RR HPBA model as shown in Figure 3c. This was due to the influence of the RR pattern in transonic flow. Based on Figure 3d, it is predicted that lift could be increased between the range 2.98% and 5.41% in the transonic flow regime.

### 4.2. Viscous Drag

**Figure 4:**
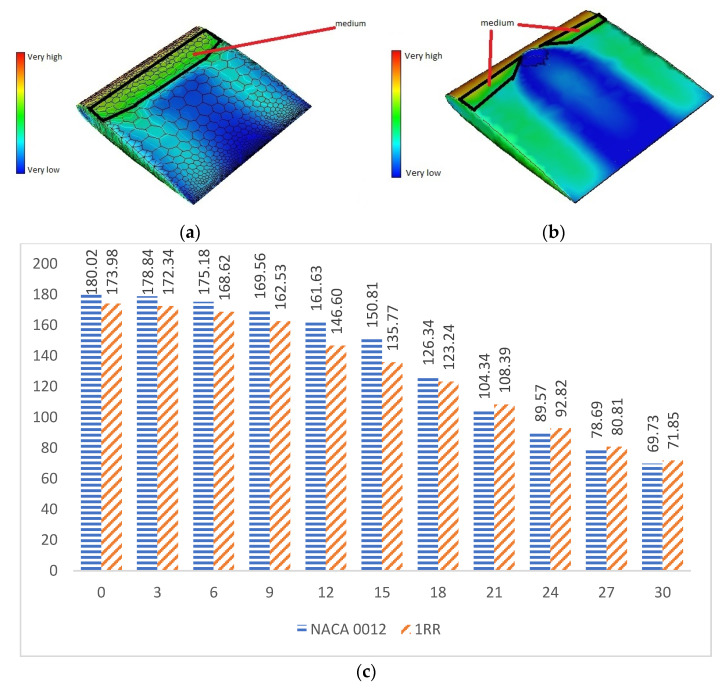

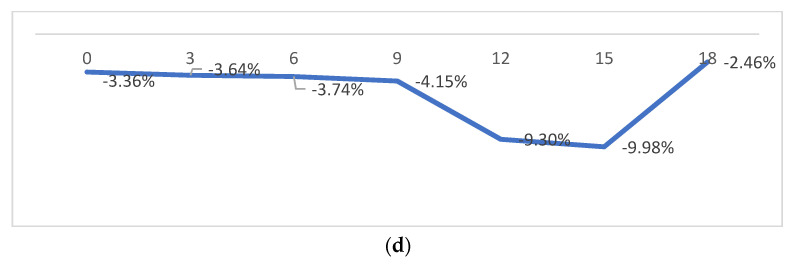
Viscous analysis. (**a**) Skin friction contour of NACA 0012. (**b**) Skin friction contour of 1RR HPBA. (**c**) Viscous generated at all angles of attack. (**d**) Viscous drag in percentage.

Figure 4a clearly shows the distribution of skin friction on the top surface of NACA 0012 aerofoil. This shows that NACA 0012 has medium values of pressure near the leading edge. Generally, an aerofoil requires lower values of skin friction as indicated in Figure 4b in order to perform effectively. The larger concentration of medium values of friction were reduced to a shorter length with the use of the RR pattern. At low and medium angles of attack i.e., from 0° to 18°, the values of viscous forces are higher in NACA 0012 when compared to 1RR HPBA as shown in Figure 4c. This was due to the influence of the RR pattern in transonic flow. Based on Figure 4d, it is predicted that viscous forces could be reduced between the range of 3.36% and 9.98% in the transonic flow regime.

### 4.3. Influence of RR Pattern in Transonic Flow

**Figure 5:**
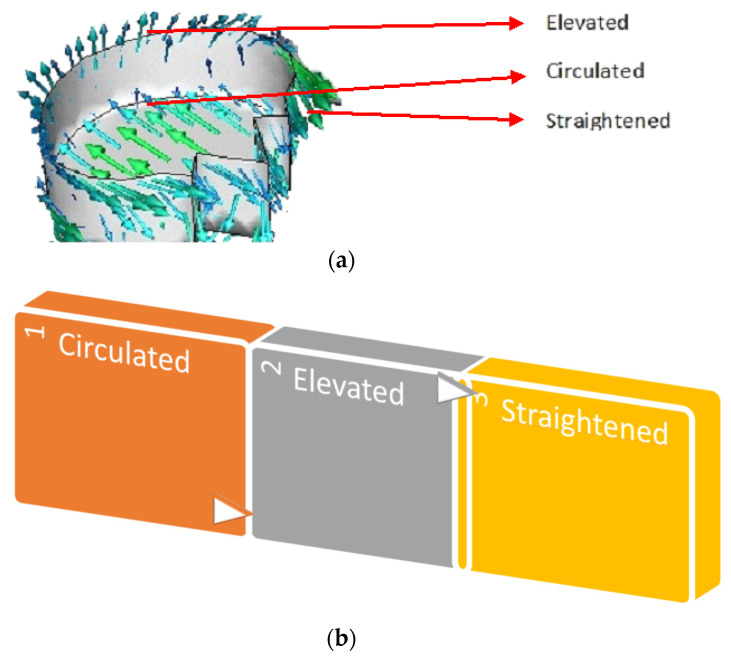
Fluid interaction. (**a**) Velocity vectors within the pattern. (**b**) Fluid flow sequence.

At higher angles of attack, the fluid flow over an aerofoil reverses due to an increase in the adverse pressure gradient. RR pattern worked well with the reversed flows. When the airflow reversed, fluids entered the pattern through its tip way and reached the head of the RR pattern as shown in Figure 5a,b. These fluids circulated and re-reversed within the hollow section and elevated to the top surface. Thus, this straightened the fluid which helped it to flow along the normal direction. Due to the elevation of fluids above the top surface, the skin friction was reduced. Furthermore, the lift was increased due to the cultivation of disturbances within the hollow section of the fluid flow. These physical alterations in the fluid influenced by the RR pattern enabled the 1RR HPBA model to produce more lift and less viscous drag at many angles of attack.

## 5. Validation

The computational results of NACA 0012 aerofoil at 0° angle of attack were validated against the experimental values [19] with the help of a grid refinement test. The mesh element size was varied from 0.5 to 1.5 as shown in the Table 1 and the obtained results were noted. The lift and drag values at 0.5 mesh size were in close agreement with that of the experimental values. This confirmed the accuracy of results and the same mesh size was used in all the calculations.

## 6. Conclusions

The hollow patterned biomimetic aerofoil showed better results when compared to conventional NACA 0012 aerofoil. In this research project, shark skin was designed, implemented, and analyzed successfully with the help of computational software. The newly developed RR pattern showed improved results in transonic flow. This research has proved that

(i) lift force could be increased up to 5.41% at 21° angle of attack,
(ii) viscous drag could be reduced up to 9.98% at 15° angle of attack,
(iii) developed a new biomimetic pattern called Raw Riblet,
(iv) surface with a little roughness is better than a smooth surface.

This research may motivate researchers in order to find more solutions from nature. Moreover, the appropriate use of a larger hollow pattern would make the aircraft maintenance work easier when compared to the current shark skin replications. The use of this pattern would enhance fuel efficiency at high maneuvering angles. Although the lift and viscous values are improved at several angles of attack, total drag problems at low angles of attack still need to be addressed. However, the aim of this project has been achieved. Future investigations on the raw riblet pattern need to be carried out with high end facilities in order to solve the problems in supersonic flow. This research emphasizes that rough surfaced designs are better and the same need to be focused on in order to enhance air transport vehicles.

## Figures and Tables

**Table 1:** Grid refinement test.

Element Size	Drag Coefficient
0.3	0.019
0.4	0.019
0.5	0.019
0.6	0.019
0.7	0.019
0.8	0.203
0.9	0.205
Consistent Value	0.019

## Abbreviations

HPBA	Hollow Patterned Biomimetic Aerofoil
NACA	National Advisory Committee for Aeronautics
RR	Raw Riblet
SEM	Scanning Electron Microscope
